# Fast Energy Storage of SnS_2_ Anode Nanoconfined in Hollow Porous Carbon Nanofibers for Lithium‐Ion Batteries

**DOI:** 10.1002/advs.202306711

**Published:** 2023-12-02

**Authors:** Fanghua Liang, Huilong Dong, Jiamu Dai, Honggang He, Wei Zhang, Shi Chen, Dong Lv, Hui Liu, Ick Soo Kim, Yuekun Lai, Yuxin Tang, Mingzheng Ge

**Affiliations:** ^1^ School of Textile & Clothing Nantong University Nantong 226019 P. R. China; ^2^ Faculty of Textile Science and Technology Shinshu University Tokida 3‐15‐1 Ueda Nagano 386‐8567 Japan; ^3^ School of Materials Engineering Changshu Institute of Technology Changshu 215500 P. R. China; ^4^ Institute of Applied Physics and Materials Engineering University of Macau Macau 999078 P. R. China; ^5^ Department of Biomedical Sciences City University of Hong Kong Hong Kong 999077 P. R. China; ^6^ College of Chemical Engineering Fuzhou University Fuzhou 350116 P. R. China

**Keywords:** charge carrier transfer, hollow porous carbon nanofibers, lithium‐ion batteries, SnS_2_ nanosheets, ultrahigh charging rates

## Abstract

The development of conversion‐typed anodes with ultrafast charging and large energy storage is quite challenging due to the sluggish ions/electrons transfer kinetics in bulk materials and fracture of the active materials. Herein, the design of porous carbon nanofibers/SnS_2_ composite (SnS_2_@N‐HPCNFs) for high‐rate energy storage, where the ultrathin SnS_2_ nanosheets are nanoconfined in N‐doped carbon nanofibers with tunable void spaces, is reported. The highly interconnected carbon nanofibers in three‐dimensional (3D) architecture provide a fast electron transfer pathway and alleviate the volume expansion of SnS_2_, while their hierarchical porous structure facilitates rapid ion diffusion. Specifically, the anode delivers a remarkable specific capacity of 1935.50 mAh g^−1^ at 0.1 C and excellent rate capability up to 30 C with a specific capacity of 289.60 mAh g^−1^. Meanwhile, at a high rate of 20 C, the electrode displays a high capacity retention of 84% after 3000 cycles and a long cycle life of 10 000 cycles. This work provides a deep insight into the construction of electrodes with high ionic/electronic conductivity for fast‐charging energy storage devices.

## Graphical abstract

The hollow porous carbon nanofiber encapsulated SnS_2_ nanosheets are designed via a simple one‐step sulfidation process between coaxial electrospinning and carbonization. The electrode exhibits a high specific capacity of 1935.50 mAh g^−1^ at 0.1 C and even 289.60 mAh g^−1^ at 30 C. Furthermore, it exhibits a stable performance with 84% capacity retention at 20 C even after 3000 cycles.

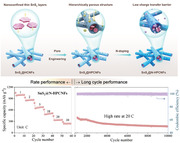

## 1 Introduction

The rapid development of electric vehicles and portable electronic devices holds a growing demand for high energy/power density lithium‐ion batteries (LIBs) with fast charging capacity.^[^ ^1^, ^2^, ^3^, ^4^ ^]^ Conventional materials with limited energy density can hardly meet such demands due to the increased charge‐transfer limitations and high resistance in thicker electrodes.^[^ ^5^, ^6^, ^7^ ^]^ One promising strategy is the employment of conversion‐typed (i.e., MoS_2_, SnS_2_) and alloy‐typed (i.e., Si, Ge) high‐capacity materials to reduce the volume of the electrodes.^[^ ^8^, ^9^, ^10^, ^11^, ^12^, ^13^, ^14^ ^]^ For instance, layered transition metal dichalcogenides SnS_2_ with high theoretical capacity (≈1136 mAh g^−1^) and large layer distance (0.59 nm) are considered an ideal anode material.^[^ ^15^, ^16^, ^17^ ^]^ However, their huge volume expansion during Li‐ion insertion/extraction usually causes pulverization of active materials, structural collapse, and continuous growth of solid electrolyte interface (SEI), resulting in rapid capacity fading.^[^ ^18^, ^19^, ^20^ ^]^ Moreover, their low intrinsic electronic conductivity with sluggish reaction kinetics can only empower LIBs with limited rate performance.^[^ ^21^, ^22^ ^]^ Therefore, the rational design of electrodes using high‐capacity materials to enable fast charge‐transfer capability is essential for fast‐charging LIBs.^[^ ^23^, ^24^, ^25^, ^26^, ^27^, ^28^ ^]^

Recently, a plethora of efforts have been made to boost the performance of SnS_2_‐based high‐capacity materials.^[^ ^29^, ^30^, ^31^ ^]^ For instance, by nanostructure strategies, SnS_2_ nanoparticles and nanosheets would shorten the ion diffusion pathway and exhibit strain relaxation to volume changes.^[^ ^32^, ^33^, ^34^, ^35^ ^]^ However, these electrodes still suffer from the inherent fragile feature and the continuous SEI growth due to the large specific surface area.^[^ ^36^, ^37^, ^38^ ^]^ In contrast, various SnS_2_‐based nanocomposites such as SnS_2_/graphene composites, core‐shell SnS_2_ nanoparticles, and SnS_2_ embedded in hollow carbon nanofibers have been rationally constructed to accommodate the mechanical strain and facilitate stable SEI formation.^[^ ^39^, ^40^, ^41^, ^42^, ^43^ ^]^ However, insufficient space or excessive space would destroy the carbon layer and cause low energy density. The incorporation of conductive fillers such as reduced graphene oxide and carbon nanofibers would improve the electronic conductivity, buffer the expansion/contraction of SnS_2_ nanosheets, and suppress particle agglomeration.^[^ ^44^, ^45^, ^46^, ^47^, ^48^ ^]^ Nevertheless, such hybrid electrodes displayed poor long‐term cycling performance due to the blocked ion diffusion pathways in graphene or carbon nanofibers. Therefore, it is still quite challenging to simultaneously achieve fast ions and electron migration in SnS_2_‐based electrodes.

In this work, hollow porous carbon nanofiber encapsulating SnS_2_ nanosheets composited electrodes (SnS_2_@N‐HPCNFs) with rapid charging, large capacity, and long lifetime were developed by a combination of electrospinning, carbonization, and sulfidation techniques. The one‐dimensional (1D) internal hollow space accommodated the huge volume changes of SnS_2_ nanosheets, while the external porosities on its surface facilitated rapid ion diffusion. Meanwhile, the 3D N‐doped carbon nanofiber networks not only provided fast electron transfer pathways, but also reduced the charge carrier transport carrier, and enhanced Li‐ions adsorption at the SnS_2_@N‐HPCNFs heterointerface. Besides, the few‐layered SnS_2_ nanosheets enhanced the reaction kinetics and made pseudocapacitive contributions. Therefore, SnS_2_@N‐HPCNFs electrodes exhibited a high specific capacity of 1935.50 mAh g^−1^ at 0.1 C and excellent rate capability ranging from 1 C to 30 C. Furthermore, it exhibited ultrahigh‐rate performance of 289.6 mAh g^−1^ at 30 C (≈19.20 A g^−1^) and exhibited superior cycling stability with over 84% Coulombic efficiency even after 3000 cycles at 20 C. Our design principle is promising to construct high‐capacity electrode materials for fast‐charging energy storage devices.

## 2 Results and Discussion

**Figure 1:**
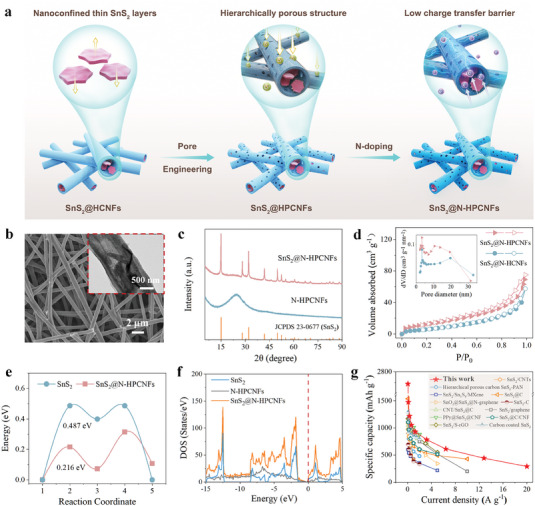
a) The schematic illustrations of SnS_2_@N‐HCNFs. b) SEM and TEM (inset image) of SnS_2_@N‐HPCNFs. c) XRD of SnS_2_@N‐HCNFs and N‐HCNFs. d) N_2_ adsorption–desorption isotherms and pore size distribution of SnS_2_@N‐HPCNFs and SnS_2_@N‐HCNFs. e) Reaction coordinate of SnS_2_@N‐HCNFs and SnS_2_. f) Total density of states of SnS_2_, N‐HCNFs, and SnS_2_@N‐HCNFs. g) A comparison of rate capability in this work with previously reported SnS_2_‐based anodes in LIBs. References: SnS_2_/CNT,^[^ ^49^ ^]^ Hierarchical porous carbon‐SnS_2_‐PAN,^[^ ^50^ ^]^SnS_2_/Sn_3_S_4_/MXene,^[^ ^51^ ^]^ SnS_2_@C,^[^ ^52^ ^]^ SnO_2_@SnS_2_@N‐graphene,^[^ ^53^ ^]^ SnS_2_@C,^[^ ^54^ ^]^ CNT/SnS_2_@C,^[^ ^55^ ^]^ SnS_2_/graphene,^[^ ^56^ ^]^ PPy@SnS_2_@CNF,^[^ ^57^ ^]^ SnS_2_@C/CNF,^[^ ^58^ ^]^ SnS_2_/S‐rGO,^[^ ^59^ ^]^ Carbon‐coated SnS_2_.^[^ ^60^ ^]^

The stability of the electrode structure and SEI are both of great importance for lithium‐ion batteries. During the lithiation/delithiation process, the electrode would experience a very pronounced phase change and huge volume change, which causes the cracks with the lithium ions consumed to form new SEI layers on the newly exposed interfaces. For instance, bare SnS_2_ nanosheets completely wrapped in a thick SEI layer were completely crushed during repeated cycles. Confined SnS_2_ nanosheets in hollow carbon nanotubes (SnS_2_@HCNFs) provide buffered space for the volume expansion of SnS_2_ nanosheets, however, the prolonged transmission paths for lithium ions increase the electrochemical resistance that limits the electrochemical performance. Therefore, pore engineering has become particularly important. The hierarchically porous structure can shorten the transmission path of lithium ions and reduce the transmission energy barrier (SnS_2_@HPCNFs) (**Figure** **1**a). Through the preparation of SnS_2_ nanosheet confined in nitrogen‐doped hollow porous carbon nanofibers (SnS_2_@N‐HPCNFs) with mixed ion and electron coupling transport synergistic, which facilitates forming a stable SEI layer and exerted to further optimize the electrode performance. On this basis, the SnS_2_@N‐HPCNFs electrode exhibited 3D interconnected networks with an average diameter of ≈560 nm by coaxial electrospinning, carbonization, and sulfidation technique, which effectively inhibits the volume expansion, and accelerates ion/electron transport (Figure 1b; Figure S1, Supporting Information). A group of parallel lattice fringe with a spacing distance of 0.19 and 0.59 nm, corresponding to the (001) and (003) plane of SnS_2_ (Figure S2a, Supporting Information). Furthermore, the corresponding Fast Fourier Transform (FFT) was shown in Figure S2b (Supporting Information), which reveals a set of obvious diffraction rings were assigned to (110), (002), and (001) crystal planes of SnS_2_ phase (JCPDS NO. 23–0677).

In addition, the energy‐dispensive X‐ray spectroscopy (EDX) mapping of the SnS_2_@N‐HPCNFs electrode indicated the uniform distribution of C, N, O, Sn, and S elements in the electrode, which illustrated that SnS_2_ nanosheet was completely confined into the 1D carbon nanofibers (Figure S3, Supporting Information). The crystal structure of the SnS_2_@N‐HPCNFs and N‐HPCNFs electrode were investigated by X‐ray diffraction (XRD) (Figure 1c). The diffraction peaks that appeared at ≈15°, 28.1°, and 30.2° were corresponded to the crystal of SnS_2_ (JCPDS NO. 23–0677).^[^ ^61^ ^]^ Furthermore, a small peak appears at the diffraction angle of ≈24°, indicating the amorphous feature of carbon nanofiber. The N_2_ adsorption/desorption isotherm of SnS_2_@N‐HPCNFs displayed typical type‐IV isotherms, suggesting its mesoporous structure.^[^ ^62^ ^]^ The specific surface area and pore volume of SnS_2_@N‐HPCNFs (43.20 m^2^ g^−1^, 0.12 cm^3^ g^−1^) were larger than those of SnS_2_@N‐HCNFs (20.70 m^2^ g^−1^, 0.09 cm^3^ g^−1^) (Figures S4 and S5, Supporting Information), indicating there were more active sites for boosting the electrochemical performance on SnS_2_@N‐HPCNFs (Figure 1d). Furthermore, mesopores (≈10.60 nm) on the surface of SnS_2_@N‐HPCNFs optimized the ion transport channel (inset of Figure 1d), which could effectively reduce the diffusion energy barrier of lithium‐ion and improve its diffusion kinetics. Due to the heteroatoms doping and rich pores on its surface, the electrode displayed excellent electrolyte wettability (Figure S6, Supporting Information). By confining SnS_2_ nanosheets in N‐HPCNFs and creating the porosities on its surface, the SnS_2_@N‐HPCNFs composited electrode exhibited a lower energy barrier (0.216 eV) than that of pure SnS_2_ (0.487 eV) after calculation (Figure 1e; Figures S7 and S8, Supporting Information). Meanwhile, though the pristine SnS_2_ is semiconducting, the SnS_2_@N‐HPCNFs were metallic due to the incorporation of N‐HPCNFs (Figure 1f). Therefore, these advantages endow SnS_2_@N‐HPCNFs in our work with better performance than most of the previously published works (Figure 1g).

**Figure 2:**
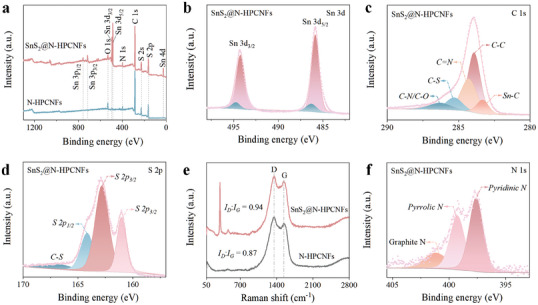
a) XPS spectra of N‐HPCNFs and SnS_2_@N‐HPCNFs. High‐resolution XPS spectra of b) Sn3d, c) C1s, d) S 2p, f) N 1s of SnS_2_@N‐HPCNFs. e) Raman spectra for SnS_2_@N‐HPCNFs and N‐HPCNFs.

In addition, the chemical composition and chemical bond states of the SnS_2_@N‐HPCNFs electrode were investigated by X‐ray photoelectron spectroscopy (XPS), as shown in **Figure** **2**a. The representative peaks of ≈284.08, 530.08, 398.08, 227.08, and 163.08 eV corresponded to C 1s, N 1s, O 1s, and S 2p for N‐HPCNFs. After adding metal elements to the SnS_2_@N‐HPCNFs electrode, the characteristic peaks of Sn 3d_3/2_ (494.08 eV), Sn 3d_5/2_ (486.08 eV), Sn 3p_1/2_ (757.08 eV), Sn 3p_3/2_ (716.08 eV), and Sn 4d (26.08 eV) appeared.^[^ ^63^ ^]^ The O element in the SnS_2_@N‐HPCNFs electrode is mainly derived from the residual oxygen‐containing functional groups on the carbon nanofibers' surface. SnS_2_@N‐HPCNFs electrode contained the two representative peaks at ≈494.20 and 485.80 eV correspond to the Sn 3d_3/2_ and Sn 3d_5/2_ of Sn^4+^, and two peaks at ≈494.70 and 486.30 eV correspond to Sn─C bond (Figure 2b).^[^ ^64^ ^]^ The existence of Sn─C bond was expected to provide a strong binding force between SnS_2_ nanosheet and carbon matrix, which can effectively prevent the aggregation and crushing of active nanoparticles during charging/discharging cycles. Figure 2c shows the high‐resolution C1s of the SnS_2_@N‐HPCNFs electrode. The four peaks at ≈283.90, 284.26, 285.29, 286.28, and 283.30 eV correspond to the C─C, C═N, C─S, C─N/C─O, and Sn─C bonds.^[^ ^65^ ^]^ The existence of C─N bond indicated that N atoms were successfully doped into the carbon matrix materials. In addition, the heteroatom doping introduces more lithium‐affinitive sites into the carbon matrix, effectively guiding the uniform nucleation of lithium and facilitating highly reversible Li^+^ insertion/extraction processes, which enhances the performance.^[^ ^66^, ^67^ ^]^ Otherwise, the S 2p peak of the SnS_2_@N‐HPCNFs electrode were resolved into four peaks (Figure 2d), which correspond to S 2p_3/2_ (161.02, 162.80 eV) and S 2p_1/2_ (164.15 eV) diffraction peaks of S^2−^ in SnS_2_.^[^ ^68^ ^]^ The C─S bond (166.84 eV) in the SnS_2_@N‐HPCNFs electrode proved an interfacial bonding between SnS_2_ and N‐HPCNFs, which was conducive to enhancing the electrochemical reaction kinetics of the composites, promoting the reaction kinetics of SnS_2_, and further enhancing its electrochemical performance. The Raman spectra of the SnS_2_@N‐HPCNFs electrode were observed as a sharp peak at 311 cm^−1^, which corresponds to the A1g mode of SnS_2_ (Figure 2e).^[^ ^69^ ^]^ The other two broad peaks at ≈1354 and 1543 cm^−1^ corresponded to the D band (defect‐related) and G band (degree of graphitization). Moreover, the ratio of SnS_2_@N‐HPCNFs electrode (0.94) was higher than that of N‐HPCNFs (0.87), revealing that more active site for the transfer and storage of Li‐ion.^[^ ^70^ ^]^ For the N 1s high‐resolution, the representative peaks at ≈397.59, 399.25, and 401.15 eV were fitted into pyridinic N, pyrrolic N, and graphitic N, respectively.^[^ ^71^ ^]^ The nitrogen content in SnS_2_@N‐HPCNFs electrodes was ≈6.2% (Figure 2a). Nitrogen‐doped hollow porous carbon nanofibers form numerous defects and vacancies on their surface, which enhance electron transport and boost lithium‐ion adsorption, thereby improving the capacity of the electrode.^[^ ^72^, ^73^ ^]^ The abundant nitrogen dopants in the SnS_2_@N‐HPCNFs electrode further improved the electronic conductivity of the composites (Figure 2f). Furthermore, the content of SnS_2_ in the composites was ≈35.10% (Figure S9, Supporting Information).

**Figure 3:**
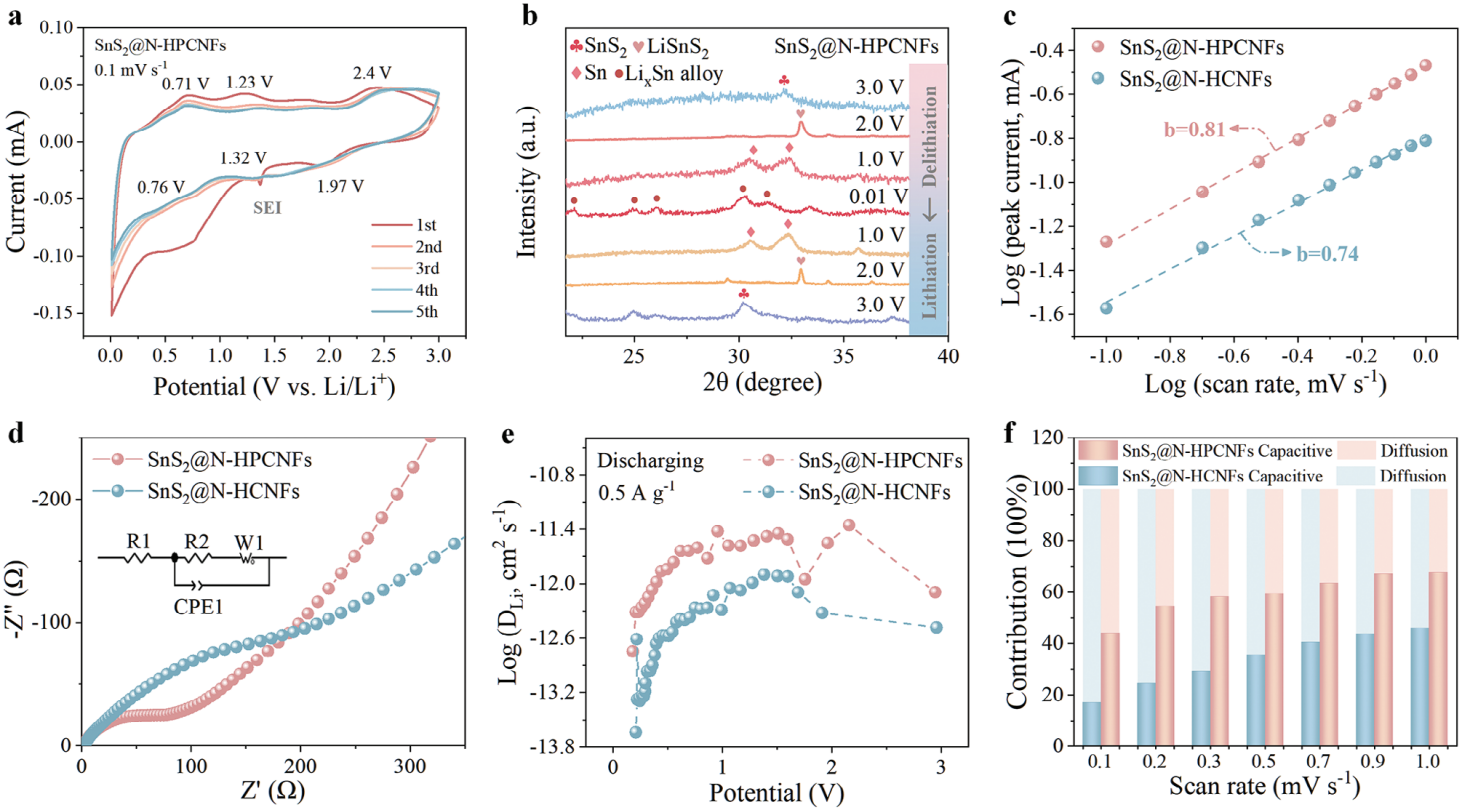
a) Initial five CV curves, and b) ex situ XRD patterns at different voltages of SnS_2_@N‐HPCNFs. c) Values of b,d) EIS spectra, e) Li^+^ diffusion coefficient at 0.5 A g^−1^ during discharging process, and f) capacitive‐controlled contribution at different scan rates of 0.1–1 mV s^−1^ of SnS_2_@N‐HPCNFs and SnS_2_@N‐HCNFs.

In order to reveal the superior electrochemical performance of the SnS_2_@N‐HPCNFs electrode during charge/discharge cycling, the electrochemical kinetics were analyzed by cyclic voltammetry (CV). The five original CV curves of SnS_2_@N‐HPCNFs at a scan rate of 0.1 mV s^−1^ with a potential range of 0.01–3 V are shown in **Figure** **3**a. During the first discharge process, the reduction peak at 1.97 V corresponded to the formation of Li_x_SnS_2_ from lithium intercalates into the SnS_2_ layers (SnS_2_ + xLi^+^ + xe^−^ → Li_x_SnS_2_).^[^ ^74^ ^]^ The small reduction peak at ≈1.32 V was attributed to the further reduction of Li_x_SnS_2_ to Sn (Li_x_SnS_2_ + (4 – x) Li^+^ + (4 – x) e^−^ → Sn + xLi_2_S). The irreversible reduction peak at ≈1.37 V was attributed to the formation of SEI film. When the voltage was further reduced to 0.76 V, the alloying reaction of Sn occurred with the continuous embedding of Li^+^ (Sn + xLi^+^ + xe^−^ → Li_x_Sn (0 ≤ x ≤ 4.4)). However, the oxidation peak at 0.71 V corresponded to the delithiation reaction of Li_x_Sn alloy during the charging process. The oxidation peaks at 1.23 and 2.40 V were attributed to the further transformation of Sn into SnS_2_. In particular, there was no peak movement and capacity loss in the subsequent cycles, indicating the stable structure and high reversibility of the composites. In order to provide further insight into the reaction mechanism of the SnS_2_@N‐HPCNFs electrode during the charging‐discharging process, ex‐situ XRD was used for analysis (Figure 3b). During the subsequent lithiation process, the characteristic peak intensity of SnS_2_ (JCPDS NO. 23–0677) gradually decreased, to be replaced by a new diffraction peak, corresponding to LiSnS_2_ phase (JCPDS NO. 22–0692). When the electrode was further discharged to 1.0 V, the LiSnS_2_ diffraction peaks gradually diminished, while the Sn diffraction peaks appeared (JCPDS NO. 04–0673), indicating further conversion to Sn. When the lithiation voltage was 0.01 V, the diffraction peak at ≈22.09°, 31.34°, 26.11° et al. corresponded to various Li_x_Sn alloy (Li_5_Sn_2_, JCPDS NO. 29–0839; Li_7_Sn_2_, JCPDS NO. 29–0837), indicated that the alloying reaction was nearly completed.^[^ ^75^ ^]^ However, the delithiation process was just opposite to the discharging process. As the charging process occurs, the Sn and SnS_2_ phases appear successively. Obviously, the ex‐situ XRD test results were consistent with that of CV, indicating the excellent reversibility of the electrode in the lithiation/delithiation process. The CV curves of the SnS_2_@N‐HPCNFs and SnS_2_@N‐HCNFs electrode at different scan rates from 0.1 to 1 mV s^−1^ are shown in Figure S10 (Supporting Information), which displayed a stable shape. Normally, the current (*i*) and scan rate (*v*) could be linked as *i = av* ^b^, where *a* and *b* were constants.^[^ ^76^ ^]^ The b values of the SnS_2_@N‐HPCNFs and SnS_2_@N‐HCNFs electrodes were 0.81 and 0.74 (by slope of log(v)‐log(i)), respectively, indicating that the pseudocapacitance characteristics of the composites (Figure 3c). Furthermore, the pseudocapacitance contribution ability of the composite electrode was further characterized according to *i = k_1_v+k_2_v* ^1/2^, where *k*_1_ represents the capacitance process and *k*_2_ represents the diffusion process.^[^ ^77^ ^]^ At the scanning rate of 1 mV s^−1^, the capacitance contribution rate of the SnS_2_@N‐HPCNFs electrode (67.57%) was higher than that of SnS_2_@N‐HCNFs (46.04%) (Figure S11, Supporting Information). Figure 3f summarizes the capacitance contribution of SnS_2_@N‐HPCNFs and SnS_2_@N‐HCNFs electrodes at different scanning rates, indicating that the SnS_2_@N‐HPCNFs electrode has an excellent capacity storage capacity. Electrochemical impedance spectroscopy (EIS) and Galvanostatic intermittent titration technique (GITT) were implemented to explore the electrode reaction kinetics. The Nyquist plots were composed of a semicircle in the high‐frequency region and an oblique line in the low‐frequency region, which represent charge transfer resistance (*R*_ct_) related to charge transfer and Warburg impedance related to ion diffusion, respectively. The *R*_ct_ of the SnS_2_@N‐HPCNFs electrode (93.60 Ω) was lower than that of SnS_2_@N‐HCNFs (324.70 Ω) after 100 charging/discharging cycles at 1 A g^−1^ (Figure 3d; Table S1, Supporting Information), which indicated the favorable effect of porous structure on electron diffusion. In addition, the $D_{Li^{+}}$ of the SnS_2_@N‐HPCNFs electrode (1.91 × 10^−12^ cm^2^ s^−1^) is higher than SnS_2_@N‐HCNFs (0.41 × 10^−12^ cm^2^ s^−1^) based on calculations, indicating that the porous structure further shortens the transmission path of lithium‐ion, reduces the ion diffusion barrier, and accelerates the diffusion of ions in the electrode (Figure 3e; Figure S12, Supporting Information).

**Figure 4:**
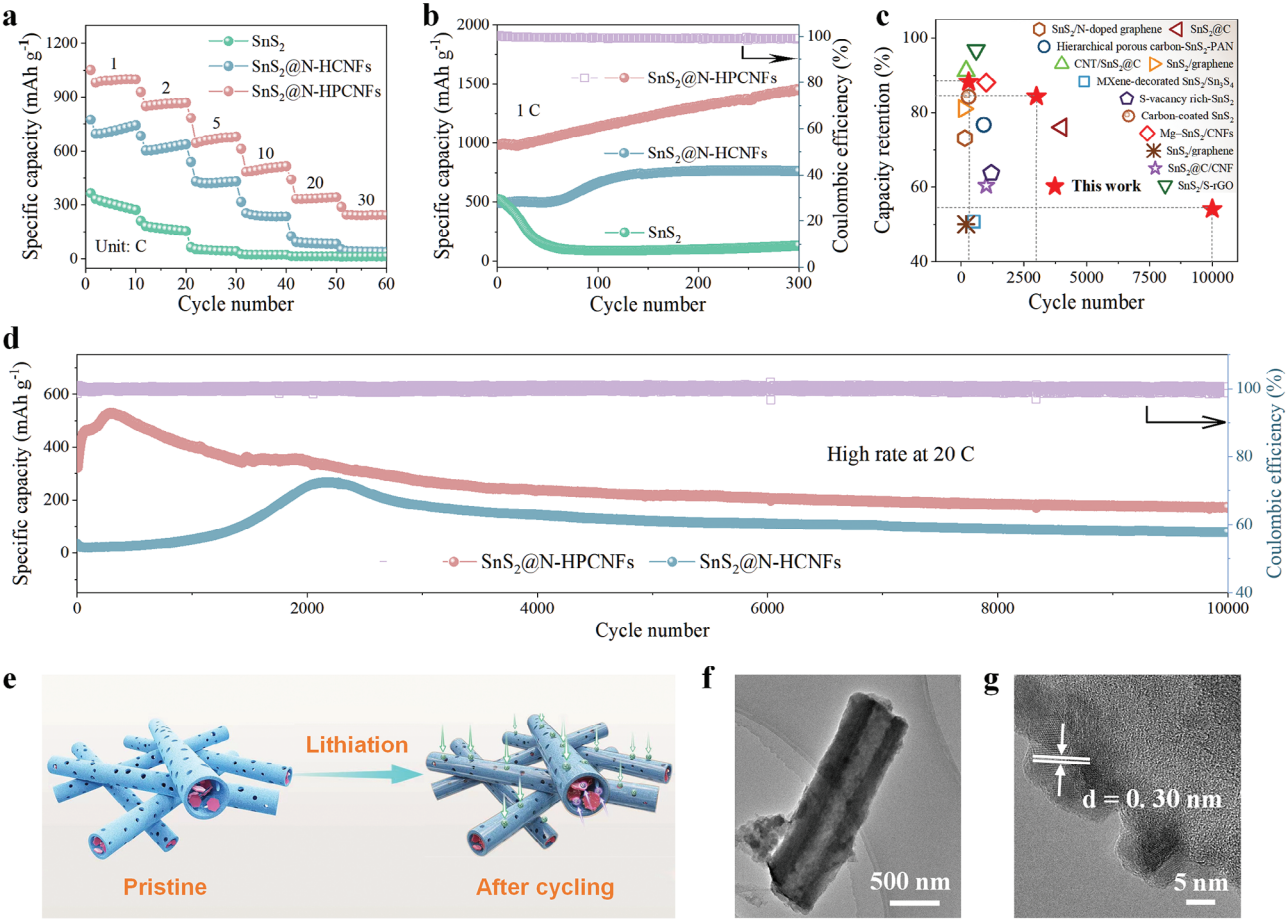
a) Rate capacity at different current densities, b) Long cycling performance of SnS_2_@N‐HPCNFs, SnS_2_@N‐HCNFs, and pure SnS_2_. c) A comparison of cycling performance with that of previously reported SnS_2_‐based anodes in LIBs. References: MXene‐decorated SnS_2_/Sn_3_S_4_,^[^ ^51^ ^]^ Hierarchical porous carbon‐SnS_2_‐PAN,^[^ ^50^ ^]^ CNT/SnS_2_@C,^[^ ^55^ ^]^ SnS_2_/S‐rGO,^[^ ^59^ ^]^ Mg‐SnS_2_/CNFs,^[^ ^79^ ^]^ SnS_2_@C,^[^ ^63^ ^]^ SnS_2_/graphene,^[^ ^82^ ^]^ SnS_2_/N‐doped graphene,^[^ ^56^ ^]^ SnS_2_@C/CNF,^[^ ^58^ ^]^ S‐vacancy rich‐SnS_2_,^[^ ^22^ ^]^ Carbon‐coated SnS_2_,^[^ ^60^ ^]^ SnS_2_/graphene.^[^ ^35^ ^]^ d) Long cycling performance of SnS_2_@N‐HPCNFs and SnS_2_@N‐HCNFs at a high current density of 20 C. e) Schematic illustration of the structure evolutions, Li‐ions and electrons diffusion pathways of SnS_2_@N‐HPCNFs. f‐g) TEM of SnS_2_@N‐HPCNFs after 500 charging/discharging cycles at 1 C.

The rate performance of SnS_2_@N‐HPCNFs, SnS_2_@N‐HCNFs, and pure SnS_2_ electrodes at different current densities ranging from 1 C to 30 C is shown in **Figure** **4**a. The SnS_2_@N‐HPCNFs electrode deliveries a high discharge capacity of 1051.80, 929.40, 784.20, 613.40, 442.30, and 289.60 mAh g^−1^ at current densities of 1 C, 2 C, 5 C, 10 C, 20 C, and 30 C, respectively. Furthermore, the SnS_2_@N‐HPCNFs electrodes still exhibited 289.60 mAh g^−1^ at a high current density of 30 C, which was much higher than that of SnS_2_@N‐HCNFs (53.90 mAh g^−1^) and pure SnS_2_ (12.30 mAh g^−1^), owing to the faster Li‐ions diffusion by porous structure and the excellent stability provided by carbon nanofibers. In addition, SnS_2_@N‐HPCNFs exhibited an ultra‐high current density of 1935.50 mAh g^−1^ at current densities of 0.1 C (Figure S13, Supporting Information). The initial charge/discharge specific capacity of SnS_2_@N‐HPCNFs were 1936.4 and 2578.0 mAh g^−1^ at 0.1 C, with an initial Columbic efficiency (ICE) of 75.11% (Figure S14, Supporting Information). Meanwhile, compared to the N‐HPCNFs (Figures S15 and S16, Supporting Information), the SnS_2_@N‐HPCNFs electrode displayed an excellent rate and long cycle performance, which related to the high conductivity provided by the N‐doped in HPCNFs. After 300 charge/discharge cycles at 1 C, the SnS_2_@N‐HPCNFs electrode still exhibited a high discharge capacity of 1446.20 mAh g^−1^ with a CE of 99% (Figure 4b). It was noteworthy that the capacity of SnS_2_@N‐HPCNFs in the initial cycles increased continuously, which was related to the electrode activation and the contribution of the stable inorganic/organic composited SEI layer.^[^ ^78^, ^79^, ^80^, ^81^ ^]^ Especially, the SnS_2_@N‐HPCNFs electrode still maintains 271.60 mAh g^−1^ at a high current density of 20 C after 3000 cycles, with a capacity retention of 84% (compared with the second discharge capacity) (Figure 4d). Even arising to 10 000 cycles, the SnS_2_@N‐HPCNFs electrode obtained a perfect capacity retention of 64% (compared with the 3000th discharge capacity), with a specific capacity of 174.20 mAh g^−1^. Moreover, it can be noticed that the long cycling performance of the SnS_2_@N‐HPCNFs electrode was far better than most of the previously published literatures about SnS_2_‐based anodes in LIBs (Figure 4c; Table S2, Supporting Information). Furthermore, due to the N‐doped hollow porous carbon framework, the SnS_2_@N‐HPCNFs electrode effectively mitigates the volume‐change‐induced strain of SnS_2_, maintains the carbon skeleton even after repeated lithiation and delithiation, and improves the transfer of ions and electrons, strong interfacial binding beneficial for stable SEI formation, which contributes to its impressive rate performance and stable cycling performance (Figure 4e). Thus, the SnS_2_@N‐HPCNFs electrode maintains the original fiber structure and no crack on the surface after 500 charge/discharge cycles at 1 C (Figure 4f,g). The XRD and XPS spectra further indicated the superior structure stability of SnS_2_@N‐HPCNFs even after 10 000 cycles at 20 C (Figures S17 and S18, Supporting Information). To further verify the application of the SnS_2_@N‐HPCNFs electrode in the Lithium‐ion full cell, which was paired with LiFePO_4_ (LFP) commercial cathode. As shown in Figures S19 and S20 (Supporting Information), SnS_2_@N‐HPCNFs//LFP electrode maintains stable specific capacities of the rate and cycling stability, which indicated that SnS_2_@N‐HPCNFs was a promising alternative to the graphite in practical applications of LIBs. The superior electrochemical performance of the SnS_2_@N‐HPCNFs electrode may result from the excellent electrochemical reaction kinetics by highly interconnected N‐doped carbon nanofiber increases the electrical conductivity, and the multi‐stage porous structure improves the Li^+^ diffusion kinetics and increases the interfacial diffusion rate.

## 3 Conclusion

In summary, the ultrafast chargeable SnS_2_@N‐HPCNFs electrode was designed via a simple one‐step sulfidation process between coaxial electrospinning and carbonization. The porous nanofibers with surface‐rich porosities optimized the ion transport channel, which can effectively reduce the diffusion energy barrier of lithium‐ion and accelerate its rapid transfer. In addition, the 1D hollow structure with large inner free space could also inhibit the large volume change of SnS_2_, which was conducive to the formation of a stable SEI layer. Moreover, the highly interconnected N‐doped carbon nanofiber networks exhibited a high conductivity, which improved the electrochemical reaction kinetics of the electrode. Therefore, the SnS_2_@N‐HPCNFs electrode exhibited a high specific capacity of 1935.50 mAh g^−1^ at 0.1 C, and even 289.60 mAh g^−1^ at 30 C. Besides, it exhibited a stable electrochemical performance with 84% capacity retention at 20 C even after 3000 cycles and a long cycle life of 10 000 cycles. This unique structural design provides theoretical guidance for the construction of high‐capacity electrode materials for fast‐charging energy storage devices.

## 4 Experimental Section

### Preparation of SnS_2_@N‐HPCNFs

SnCl_2_·2H_2_O (1.50 g) (AR, 98%, Aladdin) and PMMA (1.40 g) (polymethyl methacrylate, *M*_w_ = 15 000, Aladdin) were completely dissolved in 5.60 mL DMF (N, N‐dimethylformamide, XILONG SCIENTIFIC) under vigorously stirring for 8 h, which was used as a core solution. Similarly, 1.20 g PAN (polyacrylonitrile, *M*_w_ = 150 000, Macklin) and 0.5 g PMMA were dissolved in 8 mL DMF by stirring at 65 °C for 8 h to obtain a shell solution. For the control group, N‐HPCNFs were prepared without adding SnCl_2_·2H_2_O into the core solution by the same method, while the shell solution of SnS_2_@N‐HCNFs was without PMMA. The core–shell solution was then poured into a plastic syringe with a stainless‐steel needle at a certain spinning speed and a voltage of 15 kV for implementing the co‐electrospinning process. The as‐spun fibers were first pre‐oxidation at 250 °C for 2 h in an air atmosphere. Then, the stabilized fibers and sulfur powder (AR, ≥99.5%, Aladdin) were mixed uniformly in the porcelain boat according to the mass ratios of 1:10. The stabilized fibers were sulfuretted at 300 °C for 2 h following carbonization at 450 °C for 2 h in N_2_ atmosphere to obtain the SnS_2_@N‐HPCNFs.

### Structural Characterization

The morphology and microstructure were observed by field emission scanning electronic microscopy (SEM, ZEISS, Gemini 300) and transmission electron microscopy (TEM, FEI, Tecnai G20). The chemical valence state and composition of the composites were investigated by XPS (Thermo Scientific, NEXAS). XRD (Philips) with Cu‐Kα radiation between 5° and 90° was used to investigate the crystal structure of the composites. Raman spectra were characterized by a confocal Raman micro spectrometer (Renishaw InVia, Derbyshire, England) with a 532 nm laser source. The thermogravimetric analysis (TGA, NETZSCH STA 449 F5) was used to measure the content of SnS_2_ and carbon, which was performed from 20 to 1000 °C at a heating rate of 10°C min^−1^ in air. The pore size and specific surface of the composites was calculated by Brunauer‐Emmett‐Teller (BET) analysis.

### Electrochemical Tests

The anode electrode consisted of SnS_2_@HPCNFs, poly(vinylidene fluoride) (PVDF), and carbon black with a weight ratio of 8:1:1, which were mixed with an amount of N‐methyl‐2‐pyrrolidone and coated on copper foil and dried at 100 °C in a vacuum oven for 12 h. The mass loading of active materials was 0.4–0.5 mg cm^−2^. The CR2032 coin full cells were assembled in an argon‐filled glove box by using Li metal as the counter electrode, Celegard 2400 as the separator, and 1.0 m LiPF_6_ in EC:DMC:EMC(1:1:1 vol%) with 1.5% VC as electrolyte. As for the full cells, the SnS_2_@N‐HPCNFs electrode was activated by discharging‐charging process in a half‐cell after 10 cycles and then taken out as the anode, while matching with LiFePO_4_ (LFP) commercial cathode. Cyclic voltammetry (CV) and electrochemical impedance spectroscopy (EIS) test experiments were tested using an electrochemical workstation (CHI 660E, Chenhua). The CV test was determined on the potential of 0.01–3 V at a scan rate from 0.1 to 1 mV s^−1^, while EIS was carried out on the frequency ranging from 10^5^ to 0.01 Hz with a voltage amplitude of 5 mV. The Galvanostatic discharge‐charge (GDC) and galvanostatic intermittent titration technique (GITT) were measured under 0.01–3 V on a NEWARE battery detection system.

## Conflict of Interest

The authors declare no conflict of interest.

## Supporting information

Supporting Information

## Data Availability

The data that support the findings of this study are available in the supplementary material of this article.
