# Supplementary material for: Fast Energy Storage of SnS2 Anode Nanoconfined in Hollow Porous Carbon Nanofibers for Lithium‐Ion Batteries
Source: Adv Sci (Weinh). 2023 Dec 2;11(4):2306711. doi: 10.1002/advs.202306711 (PMC10811495; doi:10.1002/advs.202306711)
Supplement: Supplementary file 1 — Supporting Information [file ADVS-11-2306711-s001.pdf]

ADVANCED SCIENCE
Open Access

Supporting Information

for *Adv. Sci.*, DOI 10.1002/advs.202306711

Fast Energy Storage of SnS_2 Anode Nanoconfined in Hollow Porous Carbon Nanofibers for Lithium-Ion Batteries

Fanghua Liang, Huilong Dong, Jiamu Dai, Honggang He, Wei Zhang, Shi Chen, Dong Lv*, Hui Liu, Ick Soo Kim, Yuekun Lai, Yuxin Tang and Mingzheng Ge**

Supporting Information

Fast Energy Storage of SnS_2 Anode Nanoconfined in Hollow Porous Carbon Nanofibers for Lithium-ion Batteries

Fanghua Liang,† Huilong Dong,† Jiamu Dai,† Honggang He, Wei Zhang, Shi Chen, Dong Lv,* Hui Liu, Ick Soo Kim, Yuekun Lai, Yuxin Tang, Mingzheng Ge**

[*] F. Liang [+], Prof. J. Dai,[+] H. He, Prof. W. Zhang, Prof. H. Liu, Prof. M. Ge
School of Textile & Clothing, Nantong University, Nantong 226019, P. R. China
E-mail: zhangwei@ntu.edu.cn; mzge1990@ntu.edu.cn

Prof. H. Dong[+]
School of Materials Engineering, Changshu Institute of Technology, Changshu 215500, P. R. China

Prof. M. Ge, Prof. S. Chen
Institute of Applied Physics and Materials Engineering, University of Macau, Macau 999078, P. R. China

Dr. D. Lv
Department of Biomedical Sciences, City University of Hong Kong, Hong Kong 999077, P. R. China
E-mail: donglv3-c@my.cityu.edu.hk

F. Liang, Prof. I. Kim
Faculty of Textile Science and Technology, Shinshu University, Tokida 3-15-1, Ueda, Nagano 386-8567, Japan

Prof. Y. Lai, Prof. Y. Tang
College of Chemical Engineering, Fuzhou University, Fuzhou 350116, China

[+] These authors contributed equally.
Keywords: SnS_2 nanosheets; hollow porous carbon nanofibers; charge carrier transfer; ultrahigh charging rates; lithium-ion batteries

1. Supporting Notes

1.1 Lithium ionic conductivity determined by galvanostatic intermittent titration technique (GITT).

1.2 Theoretical analysis of Li-ions adsorption behavior in the electrode.

2. Supporting Figures

Figure S1. SEM image of SnS_2@N-HPCNFs.

Figure S2. a) HRTEM images and b) FTT image of SnS_2@N-HPCNFs.

Figure S3. EDX mapping of SnS_2@N-HPCNFs.

Figure S4. SEM image of SnS_2@N-HCNFs.

Figure S5. TEM image of SnS_2@N-HCNFs.

Figure S6. Contact angle of SnS_2@N-HPCNFs

Figure S7. The possible configurations of SnS_2/nitrogen-doped carbon nanofibers heterostructures (SnS_2@N-HPCNFs HSs), as well as their relative energies (ΔE) to total energy of Configuration 2.

Figure S8. Li-ion diffusion pathway in N-HPCNFs, bulk SnS_2, and SnS_2@N-HPCNFs.

Figure S9. TGA image of SnS_2@N-HPCNFs.

Figure S10. CV curves of a) SnS_2@N-HPCNFs and b) SnS_2@N-HCNFs at different scan rates from 0.1 to 1 mV s^{-1}.

Figure S11 The capacitive-controlled contribution at scan rate of 1 mV s^{-1} of SnS_2@N-HPCNFs and SnS_2@N-HCNFs.

Figure S12. GITT profile for SnS_2@N-HPCNFs and SnS_2@N-HCNFs during charging/discharging process.

Figure S13. Rate capability of SnS_2@N-HPCNFs, SnS_2@N-HCNFs and SnS_2 at 0.1-0.5 C.

Figure S14. The charge/discharge profiles of SnS_2@N-HPCNFs and SnS_2@N-HCNFs electrode at different current densities.

Figure S15. a) Rate capability, and b) long cycling performance of N-HPCNFs.

Figure S16. a, b) SEM image, c) TEM of N-HPCNFs.

Figure S17. XRD of SnS_2@N-HPCNFs before and after 10000 cycles cycling.

Figure S18. a) XPS and b) high-resolution XPS spectra of C1s of SnS_2@N-HPCNFs before and after 10000 cycles cycling.

Figure S19. a) Rate capability, and b) long cycling performance of SnS_2@N-HPCNFs// LFP.

Figure S20. Initial charge/discharge profiles of SnS_2@N-HPCNFs//LFP electrode at 0.1

C.

3. Supporting Tables

Table S1. The EIS fitting results of SnS_2@N-HPCNFs and SnS_2@N-HCNFs.

Table S2. Comparison of performance of Sn-based anode materials for Li-ion batteries.

1. Supporting Notes

1.1 Lithium ionic conductivity determined by galvanostatic intermittent titration technique (GITT)

To further study the lithium-ion diffusion properties of SnS_2@N-HPCNFs electrode, the lithium-ion diffusion coefficient was calculated by GITT. The lithium-ion diffusivity (D_{Li^+}) could be calculated by the quotation as following[1]:

$$D_{Li^+} = \frac{4}{\pi t}\left(\frac{m_B V_m}{M_B S}\right)^2\left(\frac{\Delta E_S}{\Delta E_t}\right)^2$$

Where t is the relaxation time, V_m is the molar volume, m_B is the mass loading of electrode, S is the contact area between electrode material and electrolyte, ΔE_S is the voltage change caused by pulse, and ΔE_t is the voltage change of constant current charge and discharge. In this GITT teat, the battery was discharged/charged between at 0.5 A g^{-1} for 900 s, and then relaxed under open circuit for 2 h (Figure S10).

1.2 Theoretical analysis of Li-ions adsorption behavior in the electrode.

To provide microscopic insight into the lithium storage performance of SnS_2@N-HPCNFs, density functional theory (DFT) calculations were performed by the Vienna ab initio simulation package (VASP).[2-3] The ground state ion-electron wave-functions were described by the projected augmented wave (PAW) method.[4] Electron exchange-correlation was expressed by functional proposed by Perdew, Burke and Ernzerhof within the framework of generalized gradient approximation (GGA-PBE).[5] A cutoff energy of 550 eV was adopted for the plane-wave pseudopotential. The convergence thresholds during geometry optimization were set as

10^{-4} eV and 0.01 eV/Å for total energy and residual force, respectively. The Grimme's dispersion correction with Becke-Johnson (BJ) damping functions (DFT-D3)[6] was adopted to include the weak van der Waals (vdW) interactions.

The original model of SnS_2/carbon nanofibers heterostructures (SnS_2@HPCNFs HSs) was composed of graphene 6×6 supercell and single-layer SnS_2 4×4 supercell. The vacuum slab was set to be 30 Å to avoid the periodic interactions between neighboring layers. The SnS_2/nitrogen-doped carbon nanofibers heterostructures (SnS_2@N-HPCNFs HSs) were then modeled by substituting one of the carbon atoms in graphene layer (see Figure S6). The N-doped carbon nanofiber (N-HPCNFs) was modeled by substituting one of the carbon atoms in bilayer graphene 6×6 supercell. In the meantime, bilayer SnS_2 4×4 supercell and bilayer graphene 6×6 supercell were also modeled as references. The Brillouin zone was sampled by 3 × 3 × 1 k-point meshes during structural relaxation. Denser 5 × 5 × 1 k-point meshes were applied for electronic property calculations. The climbing image nudged elastic band (CI-NEB) method [7-8] was employed to calculate the diffusion pathway and diffusion barrier of Li ion in the interlayer of HSs.

2. Supporting Figures

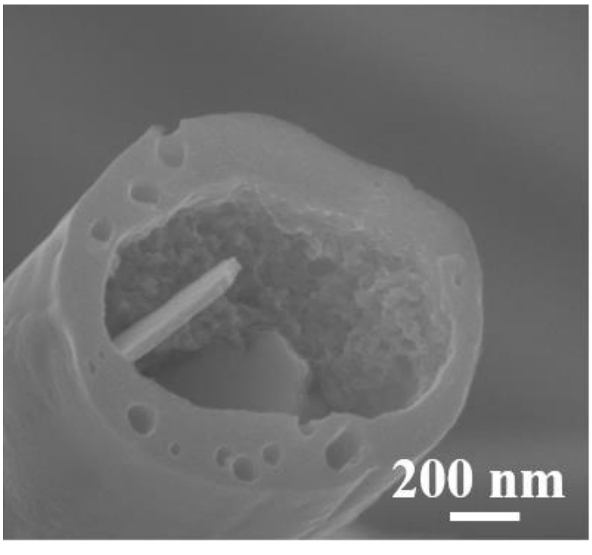

Figure S1. SEM image of SnS_2@N-HPCNFs.

Discussion: The SnS_2@N-HPCNFs electrode exhibited three-dimensional (3D) interconnected

networks with an average diameter of ~ 560 nm, which accelerates ion/electron transport. After heat treatment, the hollow structure with a wide enough diameter of about ~ 290 nm displayed large inner free space, which allows the huge volume change of SnS_2 nanosheet. Particularly, large quantities of pores on the surface of the SnS_2@N-HPCNFs electrode were beneficial for shortened the path of ion/electron transport and improved electron conductivity. It can be clearly seen that the SnS_2 nanosheet were uniformly distributed in the N-HPCNFs.

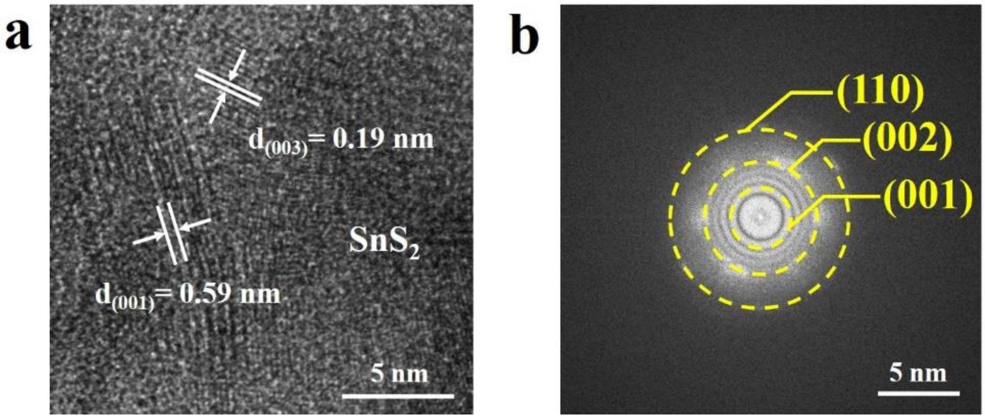

Figure S2. a) HRTEM images and b) FTT image of SnS_2@N-HPCNFs.

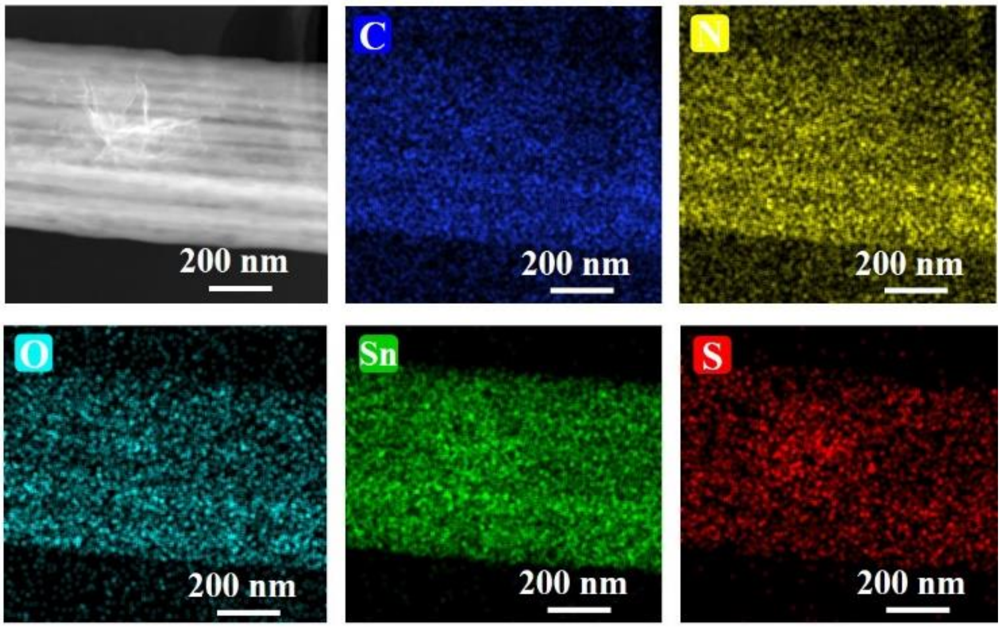

Figure S3. EDX mapping of SnS_2@N-HPCNFs.

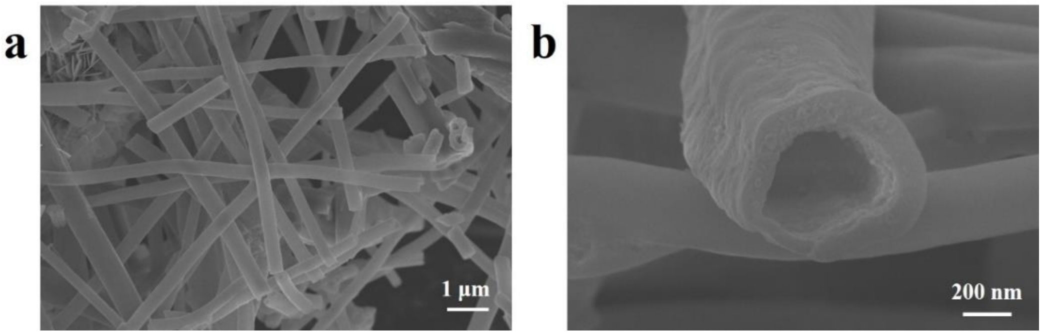

Figure S4. SEM image of SnS_2@N-HCNFs.

Discussion: The SnS_2 nanosheets embedded in N-doped hollow carbon nanofibers (SnS_2@N-HCNFs) with an average diameter of ~ 400 nm, which provide the buffered space for volume expansion of SnS_2 nanosheets, but prolongs the transmission path of lithium ions and increases the electrochemical resistance.

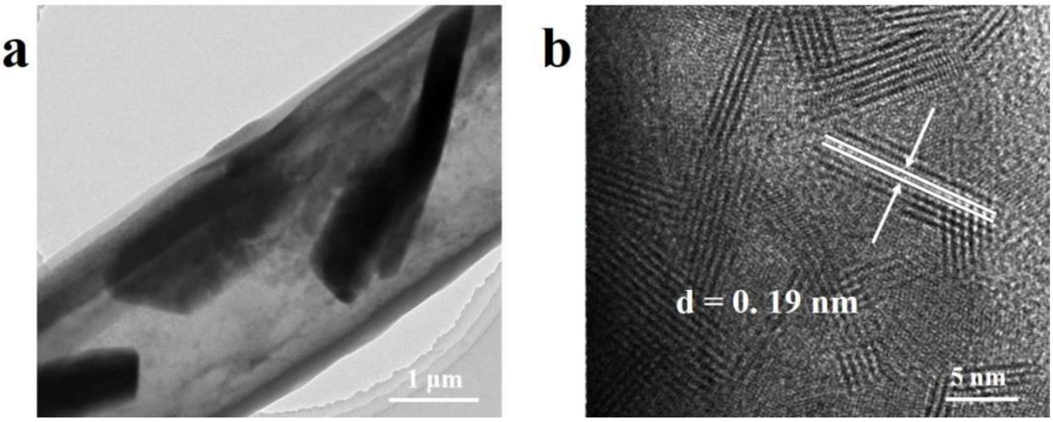

Figure S5. TEM image of SnS_2@N-HCNFs.

Discussion: A group of parallel lattice fringe of SnS_2@N-HCNFs with a spacing distance of 0.19 nm, corresponding to the (003) plane of SnS_2 (JCPDS NO. 23-0677).

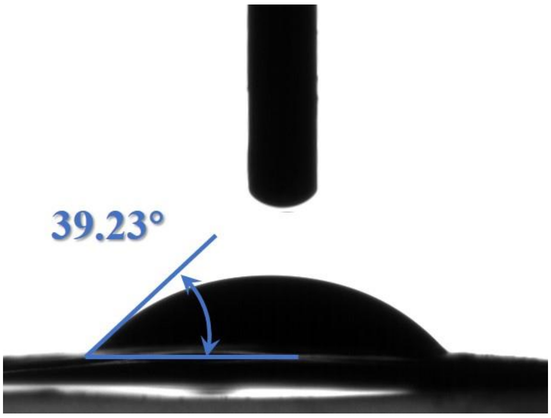

Figure S6. Contact angle of SnS_2@N-HPCNFs

Discussion: The contact angle of the electrolyte on SnS_2@N-HPCNFs was around 39.23°, showing electrolyte-philic capability. What's more, the weight of the electrode before and after adsorbing the electrolyte of SnS_2@N-HPCNFs has been calculated, which suggested per mg active materials could uptake 1.45 mg electrolyte. These evidence all demonstrated that the electrolyte could fill into the channel of N-HPCNFs.

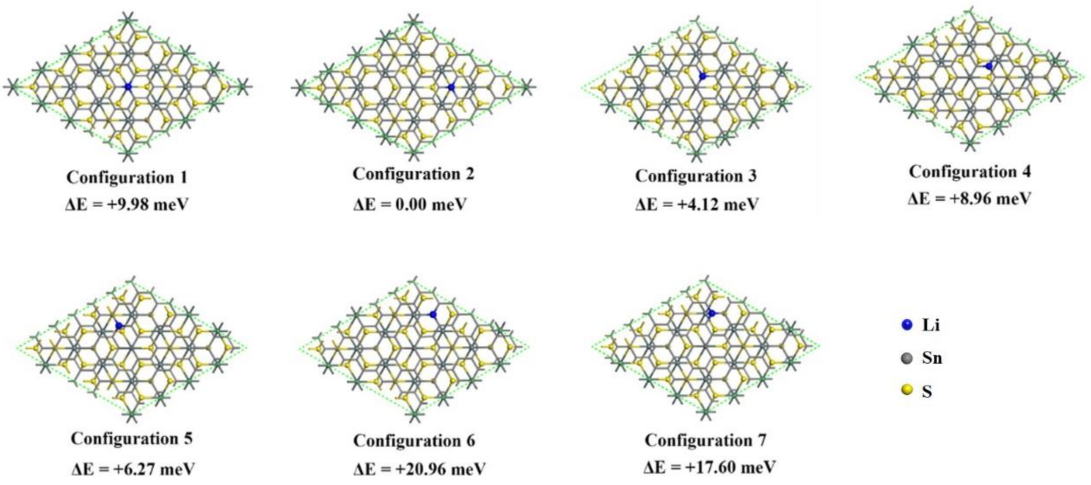

Figure S7. The possible configurations of SnS_2/nitrogen-doped carbon nanofibers heterostructures (SnS_2@N-HPCNFs HSs), as well as their relative energies (ΔE) to total energy of Configuration 2.

Discussion: To determine the most stable doping configurations of SnS_2@N-HPCNFs, we have modeled all the possible structures as shown in Figure S5. Our calculations clearly indicate that

Configuration 2 with the doped N atom on the top site of S atom in SnS_2 is the most stable configuration due to the lowest total energy. In fact, the relative energies (ΔE) do not have the formula. Relative energy is calculated as the energy difference relative to the most stable configuration among the possible configurations. The relative energy is based on the situation of Configuration 2.

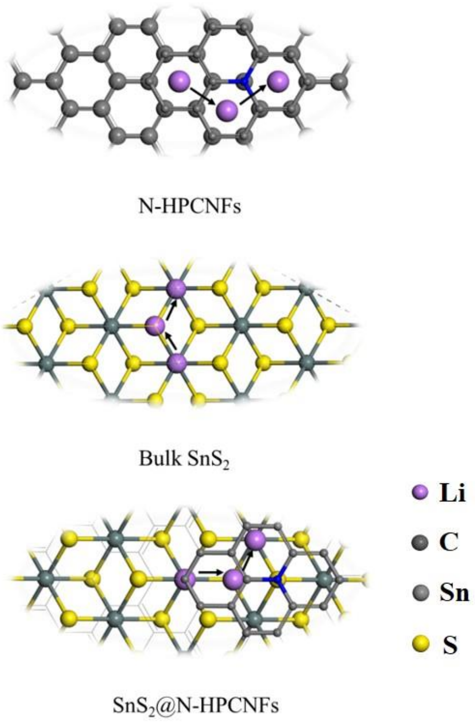

Figure S8. Li-ion diffusion pathway in N-HPCNFs, bulk SnS_2, and SnS_2@N-HPCNFs.

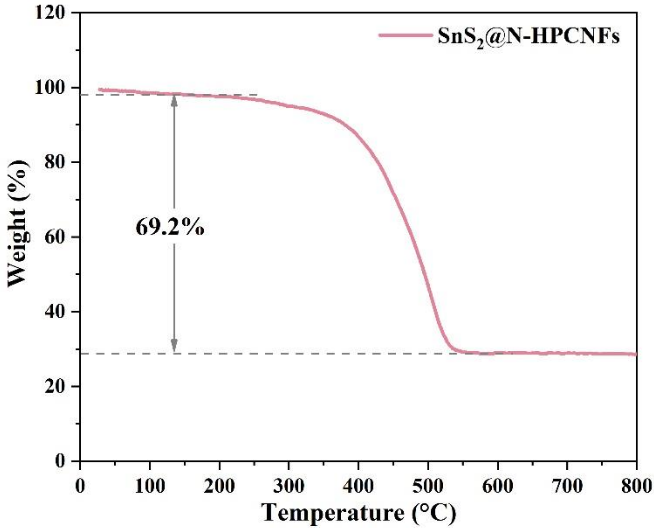

Figure S9. TGA image of SnS_2@N-HPCNFs.

Discussion: Theoretical capacity of SnS_2@N-HPCNFs.according to the calculation as following equation[9]:

$$C_{SnS_2@N-NPCNFs} = C_{SnS_2} \times wt\%_{SnS_2} + C_C \times wt\%_C = 1136 \times 35.1\% + 372 \times 64.9\% = 640.164 \text{ mAh g}^{-1}$$

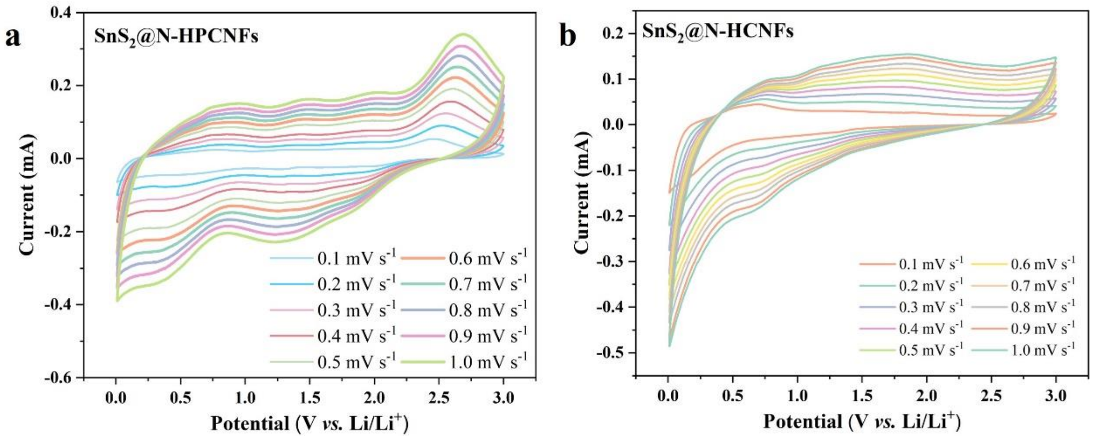

Figure S10. CV curves of a) SnS_2@N-HPCNFs and b) SnS_2@N-HCNFs at different scan rates from 0.1 to 1.0 mV s^{-1}.

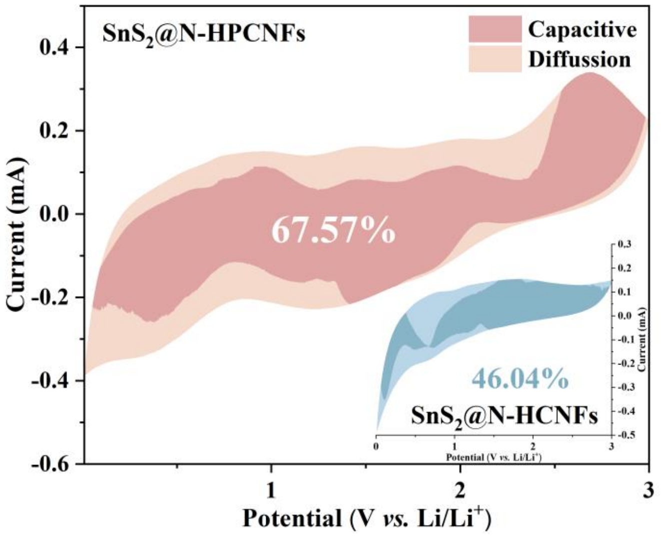

Figure S11. The capacitive-controlled contribution at scan rate of 1.0 mV s^{-1} of SnS$_2$@N-HPCNFs and SnS$_2$@N-HCNFs.

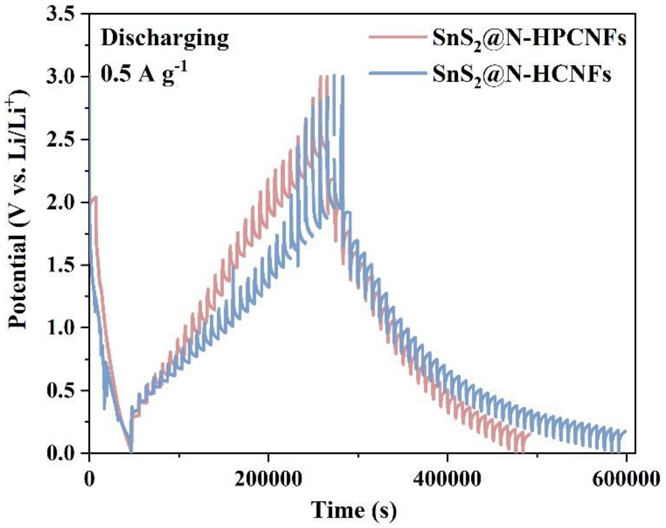

Figure S12. GITT profile for SnS$_2$@N-HPCNFs and SnS$_2$@N-HCNFs during charging/discharging process.

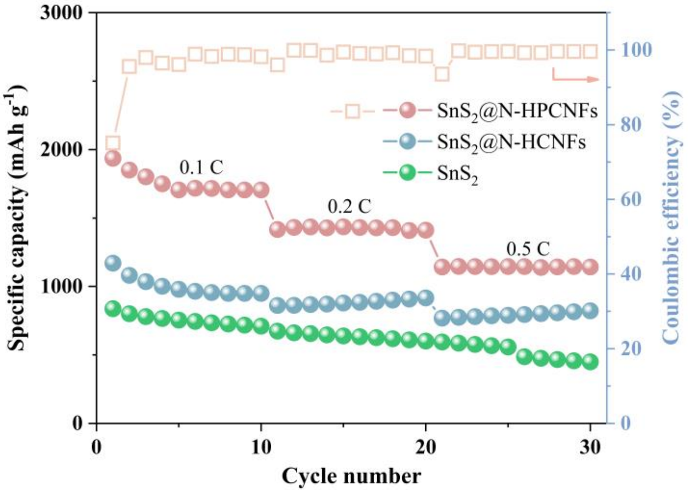

Figure S13. Rate capability of SnS_2@N-HPCNFs, SnS_2@N-HCNFs and SnS_2 at 0.1-0.5 C.

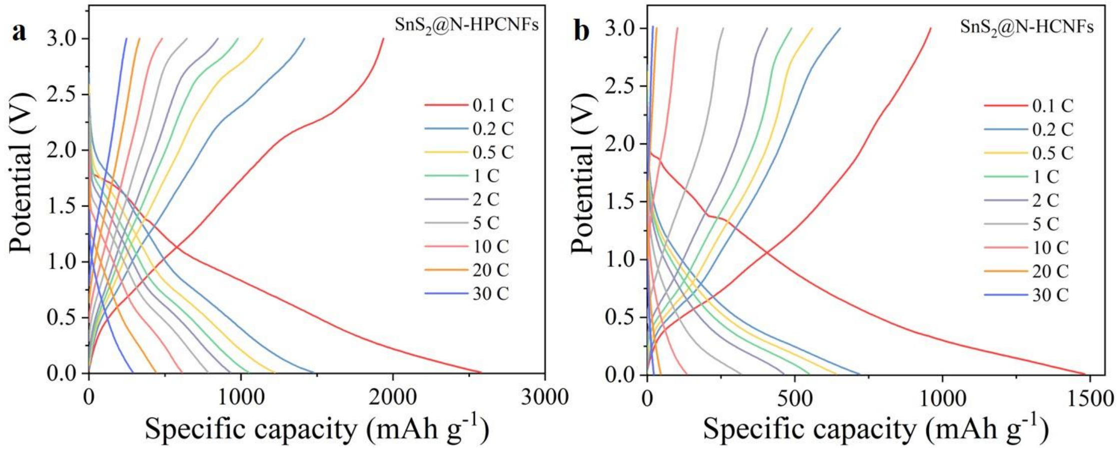

Figure S14. The charge/discharge profiles of SnS_2@N-HPCNFs and SnS_2@N-HCNFs electrode at different current densities.

Discussion: The initial discharge/charge specific capacity of SnS_2@N-HPCNFs were 1936.4, 2578 mAh g^{-1} at 0.1 C, with an initial Coulombic efficiency (ICE) of 75.11%. While the Coulombic efficiency is over than 99% at different current densities of 0.2 C, 0.5 C,1 C, 2 C, 5 C, 10 C, 20 C, 30 C, demonstrating a high reversible electrochemical reaction. However, the initial discharge/charge specific capacity of SnS_2@N-HCNFs were 1170.3, 1817.3 mAh g^{-1} at 0.1 C, with an ICE of 64.39%, which is lower than that of SnS_2@N-HCNFs.

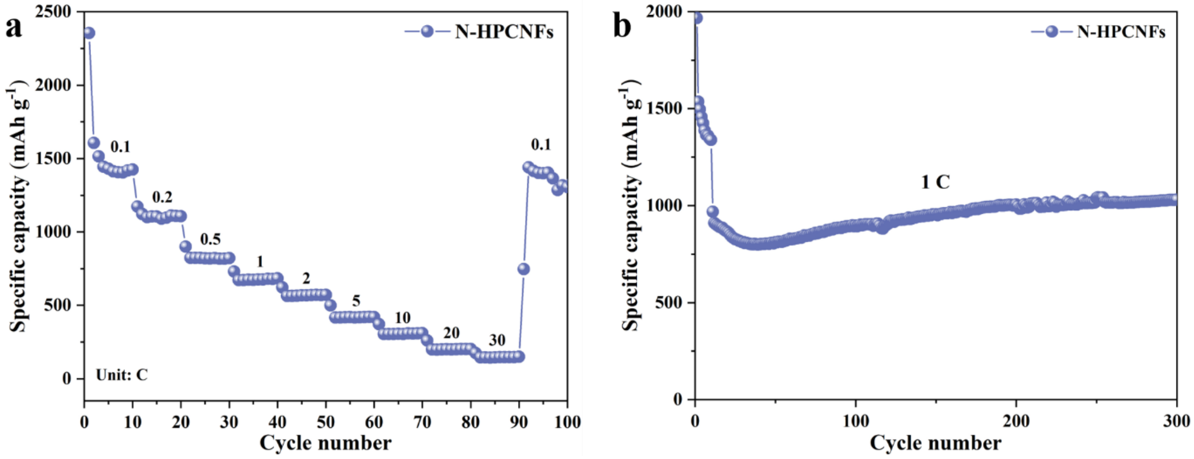

Figure S15. a) Rate capability, and b) long cycling performance of N-HPCNFs.

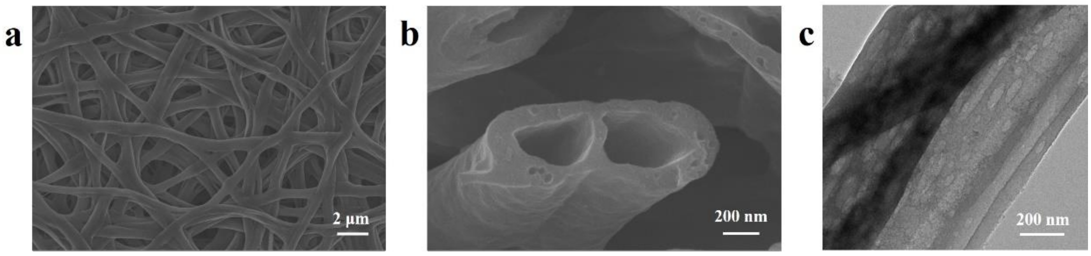

Figure S16. a, b) SEM image, c) TEM of N-HPCNFs.

Discussion: The N-HPCNFs electrode with an average diameter of ~ 579 nm is an important factor to enhance the electrode structure.

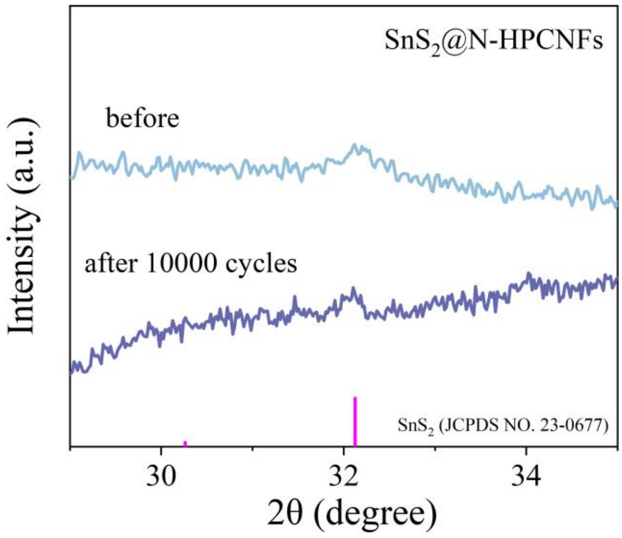

Figure S17. XRD of SnS_2@N-HPCNFs before and after 10000 cycles cycling.

Discussion: After extended cycling, the XRD spectra of the electrode approach an amorphous state, with broad and indistinct peaks, consistent with the results observed in electrodes with alloying/conversion reaction mechanisms.[10-11] The diffraction peak appearing at around 32.12°corresponded to the crystal plane of SnS_2 (JCPDS NO. 23-0677), indicating that the SnS_2 still existed after 10000 cycles.

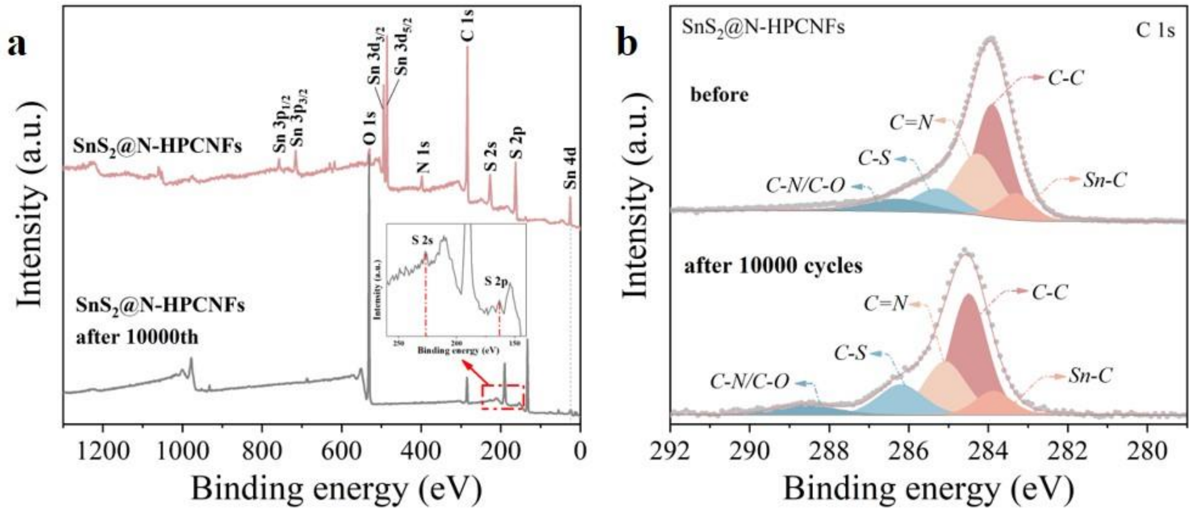

Figure S18. a) Wide XPS and b) high-resolution XPS spectra of C1s of SnS_2@N-HPCNFs before and after 10000 cycles cycling.

Discussion: The characteristic peaks at 285 eV, 531 eV, and 25.08 eV corresponded to C 1s, O 1s, and Sn 4d, respectively. Similarly, the intensities of the characteristic peaks for S 2s (227.08 eV) and S 2p (163.08 eV), were not very pronounced, likely influenced by the surface SEI film. In addition, Figure S18b gave the C 1s XPS of SnS_2@N-HPCNFs before cycling and after 10000 cycles. Before, the five peaks located at 284.50, 285.09, 286.20, 288.56 and 283.87 eV correspond to C-C, C=N, C-S, C-N/C-O and C-Sn groups, respectively. After 10000 cycling, the peaks exhibited no obvious changes, indicating high stability of SnS_2@N-HPCNFs structures, consistent with the XRD results in Figure S17.

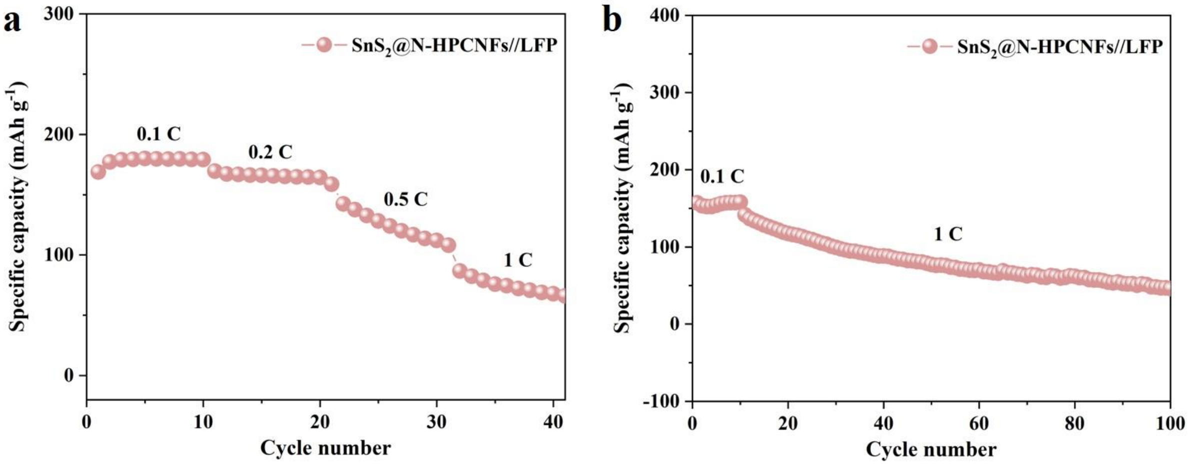

Figure S19. a) Rate capability, and b) long cycling performance of SnS_2@N-HPCNFs//LFP.

Discussion: As shown in Figure S19a, SnS_2@N-HPCNFs//LFP full cell maintains discharge specific capacities of 178.8, 169.6, 142.3, and 86.7 mAh g^{-1} at 0.1, 0.2, 0.5, and 1 C, respectively. Furthermore, the SnS_2@N-HPCNFs//LFP full cell displays a discharge capacity of 45.9 mAh g^{-1} at 1 C after 90 cycles with 0.7% capacity decay per cycle (Figure S19b), indicating superior rate capability and long cycling performance.

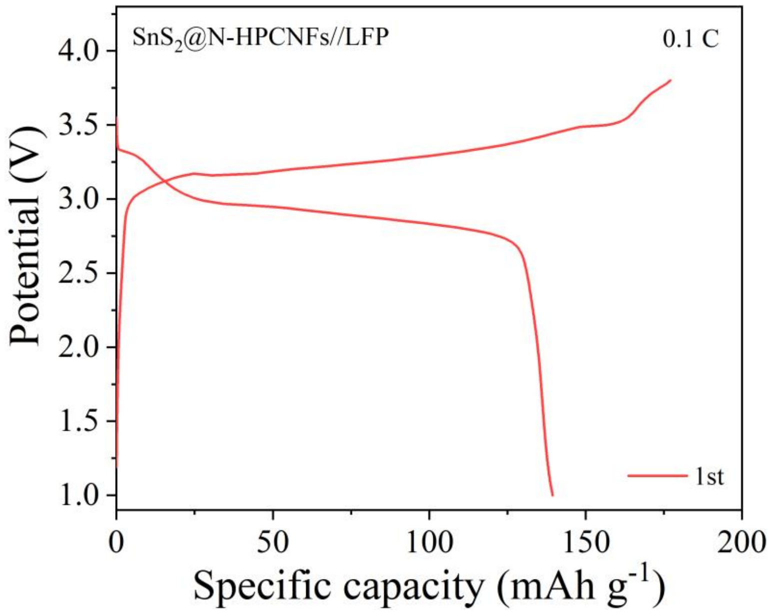

Figure S20. The initial discharge and charge capacity of SnS_2@N-HPCNFs||LFP were 139.45 and 168.86 mAh g^{-1} at 0.1 C, with a high initial Coulombic efficiency of 82.58%.

3. Supporting Tables

Table S1. The EIS fitting results of SnS_2@N-HPCNFs and SnS_2@N-HCNFs.

Material	R_s/Ω	R_{ct}/Ω
SnS_2@N-HPCNFs	1.069	93.6
SnS_2@N-HCNFs	1.034	324.7

Table S2. Comparison of performance of Sn-based anode materials for Li-ion batteries.

Sample	Current density (A g^{-1})	Cycle number	Capacity (mAh g^{-1})	Capacity retention (%)	Ref.
MXene-decorated SnS_2/Sn_3S_4	5	500	101.4	50.70	[12]
Hierarchical porous carbon-SnS_2-PAN	0.5	900	652.5	76.70	[13]
CNT/SnS_2@C	0.1	200	940	91.17	[14]
SnS_2/S-rGO	5	600	1165	96.73	[15]
Mg–SnS_2/CNFs	1	1000	532	88.06	[16]
SnS_2@C	5	4000	798.3	76.03	[17]
SnS_2/graphene	0.1	100	820	81.00	[18]
SnS_2/N-doped graphene	0.8	150	914	73.12	[19]
SnS_2@C/CNF	5	1000	483.2	60.4	[20]
S-vacancy rich-SnS_2	2	1200	765	63.75	[21]
Carbon-coated SnS_2	0.5	300	800	84.21	[22]
SnS_2/graphene	0.5	150	1050	99.00	[23]
	0.64	300	1435.6	88.21	
**SnS_2@N-HPCNFs**	12.8	3000	271.6	84.32	**This work**
	12.8	10000	174.2	54.08	

Reference

[1] C. Cao, H. Dong, F. Liang, Y. Zhang, W. Zhang, H. Wang, H. Shao, H. Liu, K. Dong, Y. Tang, Y. Lai, M. Ge, *Chem. Eng. J.* **2021**, 416, 129094.
[2] G. Kresse, J. Furthmüller, *Phys. Rev. B* **1996**, 54, 11169.
[3] G. Kresse, D. Joubert, *Phys. Rev. B* **1999**, 59, 1758.
[4] P. E. Blöchl, *Phys. Rev. B* **1994**, 50, 17953.
[5] J. P. Perdew, K. Burke, M. Ernzerhof, *Phys. Rev. Lett.* **1996**, 77, 3865.
[6] S. Grimme, J. Antony, S. Ehrlich, H. Krieg, *J. Chem. Phys.* **2010**, 132, 154104.
[7] G. Mills, H. Jónsson, G. K. Schenter, *Surface Science* **1995**, 324, 305.
[8] W. Windl, M. M. Bunea, R. Stumpf, S. T. Dunham, M. P. Masquelier, *Physical Review Letters* **1999**, 83, 4345.
[9] X. D. Li, W. Li, M. C. Li, P. Cui, D. H. Chen, T. Gengenbach, L. H. Chu, H. Y. Liu, G.

S. Song, *J. Mater. Chem. A* **2015**, 3, 2762.

[10] S. Gao, N. Wang, S. Li, D. Li, Z. Cui, G. Yue, J. Liu, X. Zhao, L. Jiang, Y. Zhao, *Angew. Chem. Int. Edit.* **2020**, 59, 2465.

[11] S. Wang, Y. Fang, X. Wang, X. W. Lou, *Angew. Chem. Int. Ed.* **2019**, 58, 760.

[12] J. F. Li, L. Han, Y. Q. Li, J. L. Li, G. Zhu, X. J. Zhang, T. Lu, L. K. Pan, *Chem. Eng. J.* **2020**, 380, 122590.

[13] Q. Hu, B. Wang, S. Chang, C. Yang, Y. Hu, S. Cao, J. Lu, L. Zhang, H. Ye, *J. Mater. Sci. Technol.* **2021**, 84, 191.

[14] B. Luo, Y. X. Hu, X. B. Zhu, T. F. Qiu, L. J. Zhi, M. Xiao, H. J. Zhang, M. C. Zou, A. Y. Cao, L. Z. Wang, *J. Mater. Chem. A* **2018**, 6, 1462.

[15] P. Zheng, Z. Dai, Y. Zhang, D. Khang Ngoc, Y. Zheng, H. Fan, J. Yang, R. Dangol, B. Li, Y. Zong, Q. Yan, X. Liu, *Nanoscale* **2017**, 9, 14820.

[16] H. Jiang, Y. Gan, J. Liu, X. L. Wang, R. G. Ma, J. J. Liu, J. C. Wang, *J J. Mater. Chem. A* **2022**, 10, 9468.

[17] Q. Sun, D. P. Li, L. N. Dai, Z. Liang, L. J. Ci, *Small* **2020**, 16, 2005023.

[18] W. Wei, F. F. Jia, K. F. Wang, P. Qu, *Chin. Chem. Lett.* **2017**, 28, 324.

[19] Y. Jiang, Y. Z. Feng, B. J. Xi, S. S. Kai, K. Mi, J. K. Feng, J. H. Zhang, S. L. Xiong, *J. Mater. Chem. A* **2016**, 4, 10719.

[20] Z. Cui, S A. He, J. Zhu, M. Gao, H. Wang, H. Zhang, R. Zou, *Small Methods* **2022**, 6, 2101484.

[21] J. Zhang, D. Cao, Y. Wu, X. Cheng, W. Kang, J. Xu, *Chem. Eng. J.* **2020**, 392, 123722.

[22] Z. Zhang, H. Zhao, Z. Du, X. Chang, L. Zhao, X. Du, Z. Li, Y. Teng, J. Fang, K. Świerczek, *ACS Appl. Mater. Interfaces* **2017**, 9, 35880.

[23] X. Li, X. Sun, Z. Gao, X. Hu, R. Ling, S. Cai, C. Zheng, W. Hu, *ChemSusChem* **2018**, 11, 1549.
